# Virtues for AI

**DOI:** 10.1007/s00146-025-02264-3

**Published:** 2025-03-06

**Authors:** Jakob Ohlhorst

**Affiliations:** Vrije Universiteit Amsterdam, Amsterdam, The Netherlands

**Keywords:** Artificial intelligence, Virtue, Artificial virtue, Artificial moral agents, Virtue epistemology, Connectionism

## Abstract

Virtue theory is a natural approach toward the design of artificially intelligent systems, given that the design of artificial intelligence essentially aims at designing agents with excellent dispositions. This has led to a lively research programme to develop artificial virtues. However, this research programme has until now had a narrow focus on moral virtues in an Aristotelian mould. While Aristotelian moral virtue has played a foundational role in the field, it unduly constrains the possibilities of virtue theory for artificial intelligence. This paper aims to remedy this limitation. Philosophers have developed a rich tradition investigating virtues, their normative domains and their structure. Drawing on this tradition, I propose a three-dimensional classification system of possible artificial virtues: virtues can be classified according to the *domain* in which virtue is an excellence, *norm* that makes a virtue an excellence, and *mode* of how the virtue delivers the excellence. With this framework, we can discern gaps in the current theorising about artificial virtues. Additionally, it gives us a tool to evaluate the competences of extant artificially intelligent systems.

## Introduction

Using virtues in the design of artificially intelligent (AI) systems has been gaining traction in recent years, notably in virtue ethics. (Coleman 2001; Stenseke 2023; Wallach & Allen 2009) This is easily explained by the fact that intelligence itself has proven to be an insufficient concept to develop a useful normative profile for cognitive competence. (Stanovich 2009) Virtue theories offer a natural way out of this conceptual deficit. This paper surveys the options for what kind of virtues AI systems could possess, where there are still gaps in theorising virtues for AI systems, and what it takes for an AI system to be virtuous.

In a way, all development of competent AI systems aims at virtues. Virtues are defined as excellent dispositions of agents, and if you aim at designing an excellent artificial agent, then you aim at designing a virtuous agent. Nevertheless, virtue approaches to AI development make more specific claims as to what kinds of competencies an AI system should possess.

This paper is not only intended as a proposal for the further development of AI virtues. The proposed virtue framework also offers a frame of reference against which we can evaluate AI systems. We can look at a particular system’s information-processing and behavioural profile, and examine whether it exhibits any kind of virtue. This allows us also to evaluate already extant AI models for their virtues, for example the currently popular machine learning models such as Midjourney or ChatGPT. For the purposes of this paper, I want to remain agnostic about what counts and what does not count as an AI system. Nevertheless, presenting virtues for AI systems posits normative constraints on what counts as a good AI system downstream.

I first introduce the theoretical options from virtue theory. We have to distinguish between *anthropic* virtues, i.e. virtues for human agents, and *artificial* virtues, i.e. virtues for AI systems. This distinction is warranted and required given the different structures of humans and artificial systems. I begin by introducing the available anthropic accounts of virtue. We can distinguish three dimensions along which virtues and the underlying theories can be classified: their *domain*, their *mode*, and their *norms*.

Second, I lay out how artificial virtues can be spelled out along these dimensions. I also review and classify extant proposals for artificial virtues within my framework. This gives us an overview of theoretical requirements for artificial virtues, and it highlights theoretical gaps in the current debate that can be fruitfully filled.

Given that our traditional, anthropic, virtues are not made for AI systems, we need to develop new virtues, designed for AI systems. This paper constitutes an initial assessment of our available virtue accounts for how they satisfy our theoretical purposes. On their basis, we will be able to design novel virtues adapted for AI systems.

## Virtues

In the broadest sense, virtues can be defined as excellent dispositions. This two-part definition goes back to the ancient Greek philosopher Aristotle (2004). Dispositions, or *hexeis*, are tendencies to behave in certain ways or bring about certain outcomes given a situation. For instance, according to Aristotle, courage is the tendency of not reacting with fear to dangerous situations.

*Dispositions* can be anchored in the agent in a myriad of ways. The *excellence* of the dispositions can also be spelled out in many ways: among others, it may be explicated as being better than others, it may be characterised as realising some values, or as fulfilling the virtuous being’s innermost function.

So, what kinds of virtues are there? I will distinguish three dimensions along which different virtues can be classified: The *domain* in which virtues operate, the *norms* which virtues realise, and the *mode* in which virtues operate. Any extant virtue can be more or less precisely classified along these dimensions, although there are mixed forms.

The different kinds of virtues that I lay out below arise from different theories of virtues. Philosophers have developed many different accounts of what makes a trait into a virtue and how this trait is structured. This gives us many competing options of what kind of thing a virtue is. For the purpose of this paper, I will take a radically pluralist stance on these different theories of virtues. If there is an available theory of how some trait is virtuous, then this constitutes a possible kind of virtue that may be interesting for evaluating or designing AI systems. You will see that these different accounts and hence possible kinds of virtues often overlap and are not mutually exclusive.

### Domain

The first, and most obvious, way how to carve up the space of virtues is by distinguishing to which domain the virtue belongs. The most prominent domain consists of *moral* virtues. When people think about virtues, they usually think about moral virtues like courage, justice and patience. This domain is defined either by the particularly moral character of the virtues that make it up or by the moral values that these virtues produce. Moral virtues explain what makes an agent a morally excellent agent.

The second domain of virtues is the *epistemic.* Epistemic virtues play an important role in Aristotelian virtue ethics because they enable and refine the moral virtues. (Aristotle 2004) They are characterised by their epistemic character, that is how they relate to epistemic practices like investigation and testimony, and by the epistemic values that they are conducive to, like knowledge and discovery. Examples of epistemic virtues are studiousness, mathematical talent, or intellectual humility. Epistemic virtues explain what makes an agent an epistemically excellent agent.

The above-mentioned are the two most-discussed and theoretically important virtue domains, but they are not the only normative domains, in which we can be virtuous. We can also be *aesthetically* virtuous; that is, be disposed toward excellent aesthetic behaviour or towards producing excellent aesthetic value (Roberts 2018). These aesthetic virtues are quite similar to epistemic virtues and include traits like creativity, endurance, or love of detail.

The last virtue domain that I want to mention is the practical, sometimes also called prudential. This is a somewhat controversial domain for virtues and excellence because the values it adheres to are purely instrumental or prudential. However, this is also the most versatile domain for what you may call a virtue. *Practical* virtue consists of the disposition to fulfil some goal, function, or purpose excellently. The typical example for such practical virtues is that the virtue of a knife is to be sharp because it cuts well. Also traits like foresight are such prudential virtues.

### Norms

Virtue accounts are also differentiated along the axis of what kinds of norms that virtues should satisfy. In other words, this is the axis specifying what the *excellence* of a virtue consists of. This is the most complex and most debated area of how we should distinguish virtues. Consequently, this will be a bit rough. Given our methodological pluralism, we can take each proposed norm for virtue to generate their own possible kind. We can distinguish two major genera of norms for virtues: agent-based virtues and value-based virtues. These, I differentiate further into particular types.

*Agent-based* virtues derive their conception of excellence from our notion of excellent agents (Slote 1995). These agent-based views can take different forms. The simplest is to take particular kinds of virtues to be primitive and basic excellences of agents. For instance, courage is just a basic and fundamental concept that we know about and to which we can hold others. Call these *virtue-first* views (Swanton 2001).

Next, there are *exemplarist* views which take the whole excellent agent—the exemplar—to be basic. On these views, we are able to recognise if someone is excellent, and we admire and emulate their traits as virtues (Zagzebski 2017). Here, what counts as a virtue derives from the moral exemplar.

Finally, there are *energetic* accounts of virtue (Hazlett 2016, p. 264). These accounts look at the agent’s capacities and functions. Virtues are the disposition of these capacities and functions to work excellently. Agents differ in their capacities, and consequently, they will differ in their virtues. For instance, we would not expect someone colour-blind to have the aesthetic virtue of skilfully matching colour palettes.

The second normative genus of virtue accounts is *value-based*. These accounts take a virtue’s excellence to lie beyond the agent *qua* agent, namely it lies in the values that virtue aims at or realises. The most popular accounts here are *Platonist* accounts of virtue. They argue that virtue is virtue insofar as how it relates to the capital-G Good going back to the work of the ancient Greek philosopher Plato (2005). The excellence of Platonist virtues consists in being guided by knowledge of the good, beautiful, and true which is the most perfect form. I take this label for any account of virtue that aims at external values even if they are not the capital-G Good, they are goods.

Another successful kind of value-based virtue account is arguably *eudaimonist*. These virtues aim at the value of the flourishing of the agent. If some trait contributes to the agent’s flourishing, then it is a virtue. There are two competing accounts of what flourishing consists in: On the one hand, there are naturalist accounts, which take the biological organism and its well-being to determine what counts as flourishing (Foot 2003; Hursthouse 2001). On the other hand, there are non-naturalist accounts that take the moral agent as the locus of flourishing (Annas 2004). This is the value-based counterpart to the agent-based energetic account.

The last value-based accounts that I want to mention are *relational* or harmony-based accounts of virtue. Instead of taking virtues to contribute to an individual’s flourishing, they take virtues’ excellence to be contributing to a community’s flourishing. These relational accounts of virtues are frequently grounded in the Confucian tradition, going back to the ancient Chinese philosopher Confucius (2010). If a trait contributes to the community’s harmony and well-being, then it is a virtue.

### Mode

The final axis along which we can distinguish kinds of virtues is by how they relate to the posited norms, or in what way the virtuous dispositions are excellent. This distinction originated in virtue epistemology but it can be extended to the other domains as well. This is the distinction between *reliabilist* virtues and *responsibilist* virtues (Battaly 2008).

Reliabilist virtues go back to Sosa’s (1980) ‘The raft and the pyramid’. They are dispositions to reliably produce true beliefs. That is, their excellence is to reliably produce the value or good of true beliefs and by doing so they guarantee our epistemic excellence. This view has been considerably refined, such that the virtues in question are now not just the disposition to reliably produce true beliefs, but also the competence to reliably succeed at something when you try. (Sosa 2015) I will go more into competence reliabilism further below.

For instance, skilful archery is a practical virtue by being the competence to reliably hit a target when you try to in the right circumstances. The relevant feature of reliabilist virtues is their reliably delivering their domain’s goods. This is not limited to epistemic or practical virtues. Driver (2004) has developed a consequentialist virtue account that can also be considered reliabilist given that it simply focusses on whether a trait delivers good outcomes or not.

Responsibilist virtues go back to Lorraine Code’s *Epistemic Responsibility* (1987) and James Montmarquet’s (1987) work. They are more inspired by Aristotelian moral virtues than by epistemological considerations about reliability. Their key element is that they are governed by good motivation. This good motivation can operate either in the virtuous dispositions’ acquisition and maintenance or in their exercise. Thus these dispositions need to be guided by their norm, but not necessarily reliably deliver them. (Baehr 2011) However, Zagzebski (1996) points out that if such dispositions do not succeed in realising the underlying good motivation then they hardly can be called virtuous.

### Three dimensions of virtue

This gives us many options on how to classify virtues according to the different accounts and the corresponding kinds. I have composed two tables which map out the three dimensions of virtue below, the first table represents reliabilist virtues, the second represents responsibilist virtues. The tables’ entries cite publications in which particular kinds of virtues have been developed. This gives you an overview of the possible options, though I do leave gaps, and to my knowledge some positions simply are not currently occupied by any theorist. These categorisations are not cut and dried; some accounts appeal to several sources of normativity or operate in several domains. However, an account’s position within the table gives you a rough idea of what kind of virtue it develops. Notably, the columns for aesthetic and practical virtues are quite deserted because few authors have worked on these issues until now.

A notable feature of reliabilist accounts is their strong prevalence towards Platonism. This is unsurprising given the structure of reliabilism: reliability is defined by its reliable production of some (external) value, traditionally truth. The easiest solution for a reliabilist virtue account is then to posit some value that the virtue produces, therefore, the most available kinds of reliabilist virtues are Platonist. However, for any value-based account it would be very easy to define a reliabilist virtue as a trait that reliably produces the values of either *eudaimonia* or harmony (Tables 1, 2).

**Table 1:** Reliabilist anthropic virtues

*Reliabilist*	Moral	Epistemic	Aesthetic	Practical
**Agent-based**				
Virtue-first	Driver (2004)		Roberts (2018)	
Exemplarist				
Energetic		Ohlhorst (2022)	Roberts (2018)	
**Value-based**				
Platonist	Driver (2004)	Battaly (2015), Greco (2010), Sosa (2015)		Sosa (2015)
Eudaimonist				
Relational

**Table 2:** Responsibilist anthropic virtues

*Responsibilist*	Moral	Epistemic	Aesthetic	Practical
**Agent-based**				
Virtue-first	Swanton (2001)	Code (1987), Montmarquet (1987)	Kieran (2012)	
Exemplarist	Zagzebski (2017)	Zagzebski (1996)		
Energetic	Anscombe (1958)	Hazlett (2016), Ohlhorst (2022)		Stichter (2018)
**Value-based**				
Platonist	Annas (2011)	Battaly (2015), Wright (2010)	Lopes (2008)	
Eudaimonist	Annas (2011), Foot (2003)		Goldie (2007)	
Relational	Confucius (2010)	Fricker (2007)		

Responsibilist accounts already cover more of the field and have developed more kinds of virtues. This shows that most of the normative distinctions that I introduced arose in the responsibilist debate. Notably, responsibilist virtue ethics is the most differentiated field, also because it is the oldest part of virtue theory. These classifications are based on a rough review of the extant literature. Arguably, there are further differentiations that would be possible depending on our theoretical interests, however, given our current purposes, this framework should suffice.

## Virtues for AI systems

Virtue theories typically rely on philosophical tradition and common sense. We all know what courage is, and since antiquity philosophers from across the world have spilled a lot of ink on what makes someone virtuous. Call these traditional virtues geared towards human agents *anthropic virtues.* AI systems, in the meanwhile, are radically different from human agents, their structures and capacities are entirely unlike ours. Consequently, we cannot simply rely on tradition to figure out what virtuous AI means. Instead, we need to develop new *artificial virtues* (Stenseke 2023), specifically designed for the purposes of describing excellent dispositions in AI systems. (Coleman 2001).

There are several kinds of virtue accounts that we need given the novel context that the presence of AI systems may create. First, we need to develop new anthropic virtues that govern our behaviour in contexts that are influenced by AI systems, especially where we design AI systems or where we interact with AI systems. Shannon Vallor calls these novel virtues *technomoral virtues* (Vallor 2016). We will set the development of these technomoral virtues aside. Instead, I will focus, second, on the artificial virtues for AI systems themselves that we need. This paper consequently engages in “robust” virtue theory for AI (Farina et al. 2022).

Virtue ethical accounts for AI systems have been suggested since at least Gips’s (2011) which originally appeared in 1995 and which relied on the thesis that connectionist models would be particularly fitting for virtue solutions. The first explicit development and formulation of virtues for AIs goes back to Coleman’s (2001), but the seminal work on AI virtue is without any doubt by Wallach and Allen (2009). They introduce the concept of the artificial autonomous moral agent (AMA) which can be virtuous by using both “bottom-up” learning processes and “top-down” explicit moral rules. By now, there have been the first implementations attempting to encode virtue in AI models, of developing “artificial virtuous agents” (Govindarajulu et al. 2019; Howard and Muntean 2017; Stenseke 2022).

These virtue ethical approaches mostly are committed to more or less Aristotelian, *responsibilist*, accounts of artificial virtue. Some explicitly include *phronesis* as a relevant virtue (Constantinescu et al. 2021; Stenseke 2023), postulating an artificial *moral agent* that makes decisions. From the traditional virtue ethicist’s perspective this will appear to be absurd: AI systems are so remote from human agency that many would be reluctant to grant agency to these models. *A fortiori* these models cannot be responsibilist and possess Aristotelian virtues.

There are two avenues here: The conservative one is to grant this point and limit future investigations of artificial virtue to reliabilist, non-agential, virtues. At least, until there are AI systems that can seriously be called agents. The more liberal avenue, which the broader debate appears to be pursuing, is to consider agency to lie more on a spectrum, on which even contemporary simple AI-models can lie. If you consider agency and responsibilist virtue to require the, however limited, ability to *weigh reasons* before taking a decision, as *phronesis* does, then some AI systems actually can do that. LIDA models would be a rudimentary example of this (Kugele and Franklin 2021). In the meanwhile, I do think that many extant AI systems do not have the resources to be responsibilistically virtuous. In what follows, I go into the three dimensions along which artificial virtue can be differentiated.

### Beyond moral virtue

Until now, accounts of artificial virtue have almost exclusively concerned the moral domain: the artificial virtue programme is about developing artificial *moral* agents and virtues. There is no artificial virtue epistemology or aesthetics. This is an obvious gap, in particular, because epistemic virtue is a precondition for moral virtue (Aristotle 2004). Additionally, while there are AI systems that make decisions with practical consequences, at the moment, most uses of AI systems are epistemic and aesthetic, not practical or moral. Consequently, this is a serious oversight. Geigel’s (2023), proposing a virtue of inventiveness, is the only properly epistemic virtue that I could come across.

Some authors are aware of this and do include epistemic aspects in their accounts. Notably, Howard and Muntean (2017) propose an account of moral knowledge for AI systems which is quite similar to Shafer-Landau’s (2003) “moral reliabilism” about act evaluation. In the meanwhile, Constantinescu et al. (2021) emphasise the importance of artificial intellectual “dianoetic” virtues—that is excellence at weighing reasons—for responsible and hence responsibilist AI systems.

However, if my original point is correct, that all AI engineering aims at creating virtuous agents because it aims at creating excellent agents, then the virtue epistemological, aesthetic, and practical perspective should be highly fruitful and a precondition for excellent artificial agency in general. Coleman (2001) does indeed suggest that most of an AI system’s virtues would be practical, that is serving the instrumental goals of their users, and not any moral purposes.

How could artificial epistemic, aesthetic, and practical virtues look then? Let us consider epistemic virtue as a case study. We have different not mutually exclusive choice points here. Is the artificial virtue responsibilist or reliabilist, i.e. does it rely on reflection and reasons or on an input–output model that is evaluated for how accurate it is? The other choice point is what constitutes the norm for classifying a disposition as a virtue. Is it the proportion of true propositions delivered? Success at answering questions or solving problems? The degree to which it resembles an exemplary researcher or manifests epistemic excellences?

Given the above-mentioned difficulties with agency, arguably reliabilist models are the easiest to implement in AI systems, also because reliability is a simple benchmark to evaluate. I will go further into responsibilism below. Concerning the values that such an artificial epistemic virtue should pursue, again the simplest case would be true propositions. A system that reliably produces true statements is already quite remarkable.

A classic example of such a reliabilist AI system would be IBM’s Watson, whose goal is to reliably diagnose cancer on the basis of clinical data (Jie et al. 2021). Note that systems like Watson are extremely domain-specific, they are only trained on data conforming to prerequisites, e.g. certain kinds of radiographies. Consequently, they may deliver arbitrary outputs on non-conform input data—say black and white photographs of the teletubbies. In other words, such systems are not sensitive to their own limitations. This means that IBM’s Watson could not be an autonomous epistemic agent. Geigel (2023) considers the same issues for the artificial epistemic virtue of the inventiveness of AI systems.

The currently popular chat implementations of large language models like ChatGPT, Bing, or Bard are known to be epistemically unreliable. More precisely, they are neither sensitive to their own mistakes, nor are their statements safely true in the sense that they wouldn’t have been made if they weren’t the case (Pritchard 2009). Large language models are aimed at reproducing linguistic patterns contained in their training data and not at being accurate. Their limited degree of epistemic reliability is a product of how linguistically plausible an output is and how accurate the linguistic training data are. This is due to the fact that the value, or goal at which large language models aim is not epistemic but linguistic.

As a consequence, large language models are at best practically reliabilistically virtuous in the narrow domain of language production. Namely, they are reliable at producing plausible-sounding text or translating a text from one language to another one. Note, however, that this artificial virtue is very advanced with large language models, they possess the writing skill of college students.

Large language models are therefore quite skilful at writing poems and imitating writing styles. Additionally, there are examples of connectionist models like Midjourney or Dall-E that are excellent at generating images. That is, these models also may exhibit aesthetic virtues. The aesthetic value delivered here seems to be something like appreciation by the average person. This excellence would result from these models’ training data which consist of images found on the internet which arguably mostly contains more generally pleasing pictures than not.

Take for instance the popular image generation model Midjourney—you give it a prompt and it delivers a nice-looking image to your specifications as an output. That is, a model like Midjourney, has the aesthetic virtue of reliably delivering generally aesthetically pleasing pictures^1^ satisfying prompts because of the aesthetically competent disposition it possesses.

While current accounts of artificial aesthetic, practical and epistemic virtues are quasi inexistent, some current AI systems appear to possess rudimentary practical and aesthetic reliabilist virtues. These rudimentary artificial linguistic and aesthetic virtues give us a great subject of study for artificial virtues, their potential and their limitations. For instance, these artificial virtues are more akin to the instrumental virtues of a knife, and not like the virtues of a knight. They are context-specific, limited to particular purposes and values, and the mentioned models are unable to develop these virtues on their own.

These reliabilist artificial virtues touch upon a related issue about the *trustworthiness* of AI. Simion and Kelp (2023) have recently offered a *functionalist* account of when an AI is trustworthy. Rejecting the idea that responsibilist virtue for AI that grounds trustworthiness is possible, they argue that an AI is trustworthy if it fulfils its function. An AI’s function can either arise through its designed purpose or its etiological history of success and use. This sounds like a reliabilist account of virtue. Carter (2023) and Nyrup (2023) criticise however that this functionalism will not entail general trustworthiness because such functions are narrow and domain-specific. At most, we may trust AI systems with representational functions that have epistemically reliabilist virtues. On a virtue-theoretical approach to the issue, we can argue that trustworthiness is at most domain-specific for extant AI systems.

### Beyond reliabilism

I just showed that a limited degree of reliabilist virtue seems to be achievable for AI systems already today. Vishwanath et al. (2023) argue similarly, that we can only expect current AI systems to perform reliably, not responsibly. Machine learning models are not responsibilist. They do not reflect or reason in any way, they are unable to plan or investigate, they cannot really solve problems (LeCun 2022). Instead, they deliver one of many statistically plausible outputs (Bender et al. 2021). If they solve a problem, it is lucky or the response is encoded sufficiently strongly in the model’s training data. If even these currently most impressive AI systems fail so miserably at possessing responsibilist epistemic virtue, then maybe full-blown responsibilist artificial virtue is still far away (cf. Simion and Kelp 2023).

However, we can soften up the requirements on what it takes to be responsibilistically virtuous and still have a useful framework at hand. Namely, Sosa’s (2015) reliabilist competence account appears to be the most fitting virtue framework for connectionist models in general. According to Sosa, a competence is defined as an agent’s disposition to succeed if they try.

Success alone, however, is not sufficient for competence or virtue. Competence follows the AAA model of Accuracy, Adroitness, and Aptness. Accuracy means that an attempt satisfies whatever value a competence aims at, adroitness means that the output is actually a product of the competence or virtue, and aptness that the attempt was accurate, i.e. successful, because of the adroit exercise of the competence (Sosa 2010). In more simple terms, a disposition is a virtue if its exercise explains its success. This framework can also be used to develop artificial AAA virtues.

Sosa’s competence reliabilism is an illustrative model for artificial virtues because it is not limited to reliable competences. Sosa expands his competence reliabilism to also include and explain responsibilist virtues: In addition to competences that reliably deliver true beliefs which constitute knowledge, there are also *auxiliary* competences that help us discover that truth (Sosa 2015, pp. 41–42).

These auxiliary competences are the responsibilist virtues according to Sosa. They serve to put the agent in a position for the reliabilist competences to function optimally, or to bring agents into contexts where their reliable competences can acquire new knowledge. For example, the auxiliary virtue of curiosity does not directly produce true beliefs, but it puts the agent into a position to acquire true beliefs. Say, by making the agent climb a mountain from which they can see everything from a new perspective.

On Sosa’s account, responsibilist virtues are the dispositions that optimise the functioning of the reliabilist virtues. This is easier to implement as artificial virtues than the full-blown agential notion of responsibilism. Artificial reliabilist faculties are highly domain-specific—recall IBM’s Watson’s limitations. In that case, auxiliary artificial virtues that shift the context in ways to optimise the reliabilist faculties’ functioning is highly valuable. AI systems do not just need basic reliability, but also auxiliary dispositions that optimise the functioning of these reliabilist virtues.

The Wikipedia plugin for ChatGPT which makes the system check its response against the relevant Wikipedia articles before outputting the response can be considered to be such an auxiliary responsibilist virtue. Again, this has nothing to do with responsibilist agency, but the plugin gives the AI system a “habit” that is auxiliary and improves the system’s (epistemic) reliability. I consider Coleman (2001) to be defending an early version of practical and moral competence reliabilism.

Some epistemologists argue that also responsibilist virtues can directly produce knowledge without the help of reliabilist virtues (Baehr 2011; Wright 2010; Zagzebski 1996). For a more full-blown account of artificial responsibilist virtue, something like the *phronesis*-based approaches mentioned above, where agents weigh reasons and evidence, would be required. Sullins (2021), for instance, argues that without the responsibilist virtue of artificial *phronesis*, and practical wisdom, we cannot consider artificial agents as moral.

Wallach and Allen (2009) go into quite some detail about bottom-up and top-down approaches to giving AI systems moral capacities. Is this the distinction between responsibilist and reliabilist virtues? It might be for humans, but not necessarily for AI systems. Let me explain: Bottom-up acquisition of a virtue means acquiring it through learning on a case-by-case basis or evolution—whether artificial or natural. This will lead to reliabilist virtues both anthropic and artificial, because no agential control is required. Bottom-up reliabilism is where connectionist frameworks excel, as I have argued.

Responsibilism and top-down approaches come apart, however. Both can be expressed in terms of implementing explicit rules and precepts like “don’t kill”, “always try to help” etc., however, top-down AI approaches and responsibilistically virtuous human agents implement these rules differently. This difference explains the shortcomings of top-down approaches for artificial virtue and the uncertain status of responsibilist artificial virtue at the moment.

Classical top-down approaches in the frame of “good old-fashioned AI” simply encode fixed explicit rules, as well as higher-order rules about which rule to follow in which situation. In the end, such a top-down AI system just strictly follows one single complex algorithm. Thereby, it becomes an inflexible input–output machine that may at certain steps seek further inputs, but nothing more. Such top-down models may exhibit reliabilist virtue, maybe even competence reliabilism, but they lack agential decision-making capacity. (*see also* Stenseke 2023).

Contrast this with how rules are implemented by a responsibilistically virtuous decision maker. If you tell and explain a rule to such an agent, they incorporate it into their deliberation without following it slavishly. When a relevant situation for the rule occurs, the agent weighs it against other relevant considerations before making a decision. Further, a responsibilist agent may be able to develop their own new rules as a guideline for the future.

Wallach and Allen (2009, p. 176) suggest that LIDA models do incorporate such decision-making processes where the system weighs different considerations. (Kugele and Franklin 2021) If we were able to communicate a new rule or reasons to such a model, or it developed its own rules, then we would have made strides towards artificial responsibilist virtue. Consequently, there is some optimism for artificial responsibilist moral, epistemic and aesthetic agents.

Ohlhorst (2022) argues that reliabilist and responsibilist virtue just designate the (energetic) excellent functioning of Type 1 and Type 2 cognition within a dual-process framework (Kahneman 2011). That is, reliabilist virtues are the dispositions of fast automatic context-specific Type 1 processes to function excellently, i.e. reliably. Responsibilist virtues are the disposition of slow, controlled domain-general Type 2 processes to function excellently. LIDA and similar models are inspired by dual-process architectures. Consequently, they are promising candidates for incorporating both reliabilist and responsibilist virtues. Indeed, Stenseke’s (2022) model incorporates analogues to both types of processes. It consequently has the potential to incorporate both kinds of virtues.

### The norms of artificial virtue

The debate over artificial virtue is most advanced concerning what norms should determine what counts as virtuous. Every type of virtue account has already been proposed implicitly or explicitly for artificial virtues. I will go through the different norms one by one.

#### Virtue first

Taking the anthropic virtues that we already know from common sense and translating them to AI systems is bound to run into many difficulties. It is too anthropocentric because AI systems are fundamentally different agents. (Stenseke 2023).

Nevertheless, it has been at least partially proposed by Berberich and Diepold (2018). Namely, they use a list of Aristotelian (2004) key virtues like justice, temperance, and courage, and examine how they might play a role and be implemented with an artificial moral agent. That is, they attempt to translate our ordinary anthropic virtue concepts into artificial virtue concepts. I would argue that we should not expect any extant or near-future AI system to exhibit the classical Aristotelian virtues. Sullins (2021) argues that an artificial analogue of *phronesis* is a precondition for artificial agents’ morality. Coleman (2001) and Vishwanath et al. (2023) also rely on catalogues of virtues from different sources.

#### Exemplarism

Exemplarism for artificial virtues has garnered considerable interest recently. The first proposal was by Govindarajulu et al. (2019) who have developed a modal logic that encodes acts and traits of agents, admiration of acts, as well as the acquisition of traits of agents whose acts are admired, i.e. moral exemplars. This permits describing and modelling an agent admiring a moral exemplar that is virtuous, as well as the agent’s acquiring the exemplar’s traits by imitating their behaviour.

Stenseke (2022, 2023) has implemented a simplified version of this mechanism in his simulation of a virtuous community, where an agent adopts another’s behavioural profile if the latter exhibits a higher eudaimonic evaluation. I will go deeper into the nature of this evaluation further below.

Finally, Kim (2021) has proposed an exemplarist approach to Wallach and Allen’s (2009) model. Namely, Kim suggests that connectionist models should be excellently suited for imitating the behaviour of moral exemplars. That is, a model is trained with an exemplar’s behavioural profile. Exemplarism seems to lend itself to applications of artificial virtue. Van Rooij et al. (2023) criticise this kind of proposal as computationally too demanding for connectionist models.

#### Energetic accounts

Energetic accounts of virtue argue that instead of taking some established notion of excellence to which the agent is held, we should look at what kind of thing the agent is. The capacities and resources of the agent tell us how it can be virtuous. In a minimal way, Wallach and Allen (2009) subscribe to this point. They argue that connectionist models are naturally suited to imitate the complex behavioural patterns that constitute virtuous behaviour. Thus, they argue that connectionist models are energetically suited to be virtuous. (*see also* Gips 2011).

Another arguably energetic account is the *moral functionalism* proposed by Howard and Muntean (2017). What is or can be virtuous or morally good supervenes “on the role of the functional and behavioral nature of the moral agent: its decision, its output state, are functional in nature, individuated by its dependence on the input, the previous output … and other, current, or previous, moral states” (Howard & Muntean 2017, p. 134). An AI system’s virtues are based on its structure and nature. Given that we are under any circumstance constrained by the artificial systems’ structure, it is natural to orient ourselves towards this structure from the outset in the development of artificial virtues. I take energetic accounts to be the most productive approach to developing artificial virtues for AI systems that exist already, given that they take their extant resources into account.

A virtue version of Simion’s and Kelp’s (2023) proposal would also be energetic. Energetic virtue accounts can offer a third alternative to etiological and design functions that they introduce. *Counterfactual functions* may be purely dispositional without ever manifesting historically or being designed for something—an AI system might for example be excellently suited for classifying minerals but no one ever has the idea to use it this way. It would still be an energetic epistemic virtue of the system.

#### Platonist virtue

Just as Platonism is the default for reliabilist epistemic virtues because it is easy to clearly define a target external value, it is also the standard approach for artificial virtues. Even approaches that appeal to other virtue theoretical models end up implementing a Platonist approach targeted at externally defined values.

The most prominent case here is Stenseke’s (2022) excellent proposal: While he formulates his proposal in terms of a eudaimonistic model, he fixes external parameters that determine what counts as an individual agent’s eudaimonistic value. His artificial agents simply strive to maximise this externally imposed “e-value” which is the Platonic good for these agents. Note that Stenseke is aware of this. Berberich and Diepold (2018) also grapple with this issue, noting that externally set reward functions often fail to realise the actual underlying value. This problem is known as “Goodhart’s law”.

Coleman (2001) also advocates for a Platonist model of artificial virtue. AI systems should strive to maximise human wellbeing. She does not think that AI systems are possible subjects of moral value and hence the only thinkable value at which artificial virtue should aim is human wellbeing. Tonkens (2012) criticises that the creation of such agents would in turn not be virtuous by the creator.

#### Eudaimonism

Eudaimonism about virtue is the claim that virtues are the traits that contribute to an agent’s flourishing given the agent’s nature. We can formulate this flourishing either in terms of happiness or in terms of biological well-being. Note that *eudaimonia* in this sense is not hedonistic, it goes beyond simple pleasure or the maximisation of one particular value—life in the experience machine or a permanent heroin drip would not mean *eudaimonia*. AI systems as we can conceive of them currently are constitutionally unable to flourish in this sense—it is unclear in what artificial flourishing should consist. Choi (2023) suggests a naturalist eudaimonist account of artificial virtue in response to Simion and Kelp (2023) where fulfilling the etiological function explains continued use and survival, but this lacks the well-being aspect.

As mentioned, Stenseke (2022) incorporates a eudaimonistic function into his AI systems. However, what it values positively is not inherent to the AI system’s nature, therefore it is Platonist. Consequently, he calls his approach *functionalist eudaimonia*. What his approach nevertheless models to a limited extent is that a virtuous agent is in a sense rewarded for their virtue by flourishing. When you act virtuously, you realise your nature as an agent or organism, and consequently you flourish, which in turn reinforces your virtuous behaviour.

#### Harmony

The last normative source for virtue has seen limited attention in artificial virtue research because it falls out of the traditional Aristotelian framework. However, there have been attempts in this direction. Harmony as a normative source for relational virtue is the communal counterpart to individualist eudaimonia. True well-being flows from the community’s thriving and not vice versa.

You might think that Coleman’s (2001) proposal goes in that same relational direction, but this is mistaken because it does not consider AI systems as a morally relevant element. Harmony *with* the AI systems is not required according to Coleman.

Liu (2022) argues on the basis of surveys about care robots that we should develop artificial relational virtues that foster harmony in inter-human and human–machine relations. Gamez et al. (2020) make a similar argument, showing that artificial agents are ascribed a reduced degree of responsibility relative to human agents, but nevertheless a degree of responsibility. Consequently, AI systems that are socially integrated need virtues that foster social harmony, if they are to be accepted. Finally, criticising Simion and Kelp (2023), Song (2023) and Nyrup (2023) argue that genuine trustworthiness of AI systems requires dispositions that foster social harmony. Ultimately, a generally trustworthy AI needs relational virtues.

## Surveying the artificial virtues

We can now recycle the tables that I introduced above to look at the field of artificial virtue. This shows immediately the total dominance of artificial moral virtue approaches over others. There is a massive gap for artificial epistemic, aesthetic, and practical virtue accounts relative to artificial moral virtue accounts. These domains are considerably underexplored. Especially, given the great promise of virtue accounts for AI systems (Tables 3, 4).

**Table 3:** Reliabilist artificial virtues

*Reliabilist*	Moral	Epistemic	Aesthetic	Practical
**Agent-based**				
Virtue-first	Coleman (2001), Vishwanath et al. (2023)			Coleman (2001)
Exemplarist	Vishwanath et al., (2023)			
Energetic		Carter (2023), Howard and Muntean (2017)		Simion and Kelp (2023)
**Value-based**				
Platonist	Coleman (2001)	Geigel (2023)		Coleman (2001)
Eudaimonist				Choi (2023)
Relational	Nyrup (2023), Song (2023)

**Table 4:** Responsibilist artificial virtues

*Responsibilist*	Moral	Epistemic	Aesthetic	Practical
**Agent-based**				
Virtue-first	Berberich and Diepold (2018), Sullins (2021)			
Exemplarist	Govindarajulu et al. (2019), Kim (2021), Stenseke (2023)			
Energetic	Gips (2011), Wallach and Allen (2009)			
**Value-based**				
Platonist	Constantinescu et al. (2021), Stenseke (2022)			
Eudaimonist	Stenseke (2022)			
Relational	Gamez et al. (2020), Liu (2022)			

What the tables also show also show, is that reliabilist approaches to artificial virtue are still underexplored. The distinction between reliabilism and responsibilism is not always clear, and the theoretical demandingness of responsibilism is often underestimated. Given its useful theoretical resources, arguably competence reliabilism (Sosa 2015) is the most promising approach to artificial virtue at the current stage of development. This is especially the case given that it also incorporates a less demanding account of auxiliary responsibilist virtue.

I also would argue that researchers on artificial virtue should reconsider the norms they aim at with their proposals. While exemplarism or virtue-first approaches may be enticing, at current levels, they only promise an artificial simulacrum of the original anthropic virtues. Platonism is valuable in its simplicity and will continue to play an important role, but energetic accounts of virtues are arguably the domain with the most potential. In the long run, relational accounts of artificial virtue will become key for aligning AI systems with human society and interests.

This gives us a framework to develop new artificial virtues. As mentioned, I would advocate looking into the development of artificial competence reliabilist energetic virtues. It is also highly urgent to expand artificial virtue research into epistemic, aesthetic, and practical domains. Note also, that we can use this framework not only for the development of new AI systems and virtues. We can also evaluate whether some particular extant AI system satisfies our requirements of excellence. For instance, I argued that a large language model like GPT is practically virtuous but epistemically highly unreliable.

## Data Availability

This paper has no associated data.

## Footnotes

1 Indeed it appears to be incapable of delivering actually ugly pictures, but will always exhibit a certain aesthetic, colour palette and composition. This has led to a sport of trying to get Midjourney to deliver ugly outputs. Obviously if it had been trained on conventionally ugly material, it would now deliver conventionally ugly outputs.
